# The Relationship between Physical Activity and Academic Procrastination in Chinese College Students: The Mediating Role of Self-Efficacy

**DOI:** 10.3390/ijerph182111468

**Published:** 2021-10-31

**Authors:** Kai Ren, Xiaolu Liu, Yujuan Feng, Changqing Li, Dingding Sun, Ke Qiu

**Affiliations:** 1 College of Physical Education and Health Sciences, Zhejiang Normal University, Jinhua 321004, China; Renkai2016@zjnu.edu.cn (K.R.); sundingding2021@163.com (D.S.); Qiuke0816@163.com (K.Q.); 2 Department of Agriculture, Food Science, and Kinesiology, Eastern New Mexico University, Portales, NM 88130, USA; 3 Department of Physical Education, Shandong University of Art and Design, Ji’nan 250014, China; fengyujuan77@163.com; 4 College of Physical Education and Health Sciences, Chongqing Normal University, Chongqing 400047, China; 20170005@cqnu.edu.cn

**Keywords:** physical activity, self-efficacy, academic procrastination, academic performance, college students, mediation analysis

## Abstract

Background: Academic procrastination (AP) has been a common problematic behavior in college students. While physical activity (PA) has been reported to increase self-efficacy and reduce AP, less is known about the potential relationships among them. Therefore, this study aimed to investigate the mediating effect of self-efficacy on the relationship between PA and AP. Methods: 687 Chinese college students (51% males, 49% females) aged 17–23 years (*M* = 19.59, *SD* = 0.89) participated in the study. PA, self-efficacy, and AP were assessed using the Physical Activity Rating Scale-3 (PARS-3), the Generalized Self-Efficacy Scale, and the Procrastination Assessment Scale-Students (PASS), respectively. Pearson correlation analysis, linear regression analysis, and mediation analysis were used to analyze the data. Results: (1) PA has a significant and negative impact on college students’ AP, (2) PA has a significant and positive impact on college students’ self-efficacy; (3) self-efficacy has a significant and negative impact on AP in college students; and (4) self-efficacy significantly mediates the relationship between PA and AP. Conclusions: PA is an effective intervention for directly and indirectly decreasing college students’ AP. Therefore, more intervention efforts should focus on the promotion of PA in higher education to improve students’ self-efficacy and thus, to reduce AP among college students.

## 1. Introduction

Academic procrastination (AP) is an intended action to postpone or delay the completion of timely academic activities unnecessarily [1,2,3,4] and is highly prevalent among college students [1,3,5,6]. It is reported that over 70% of college students regularly postpone educational tasks such as readings and writing assignments [7,8]. Moreover, over 40% of college students have self-identified as frequent procrastinators [9]. Extensive studies have explored the consequences caused by AP. Clear evidence shows that AP could lead to serious problems related to students’ studies, work, and quality of life (e.g., poor academic performance, negative mental health outcomes, and low levels of life satisfaction) [10,11,12,13,14]. Therefore, reducing students’ AP behaviors is deemed necessary and critical for achieving student success in higher education.

### 1.1. Physical Activity and AP

According to the latest edition of Physical Activity (PA) Guidelines for Americans, adults should do at least 150 to 300 min of moderate-intensity aerobic activity (or 75 to 150 min of vigorous-intensity aerobic activity) with at least 2 days of moderate- to high-intensity muscle-strengthening activity every week in order to obtain substantial health benefits [15]. Numerous physical and mental benefits of participating in regular PA have been well documented. For example, PA can reduce the risks of obesity, stroke, and overall mortality [16,17] and improve cardiovascular and musculoskeletal health [18]. Likewise, PA is beneficial to deal with stress, anxiety, and depression, as well as improve self-image and self-confidence, favoring a better perception of the quality of life [19,20,21,22,23]. Overall, PA has been widely cited as a costless and effective intervention in dealing with a variety of unhealthy lifestyle behaviors and health problems [15].

Many researchers have found a negative relationship between PA and general procrastination. Clear evidence shows that individuals who participate in regular PA (e.g., more than 150 min per week) demonstrate lower levels of procrastination because participating in PA can increase time management skills [24]. Shi et al. (2021) also specified the types of PA (i.e., light-intensity, moderate-intensity, and vigorous-intensity PA) that affect irrational procrastination and found that the greater intensity of PA, the less procrastination [7]. Similar results were reported by Zhong and Chu (2013), who examined the association between PA (time, intensity, and frequency) and general procrastination among 220 Chinese college students. The authors found that procrastination is significantly correlated to PA time and intensity. Zhong and Chu (2013) also found that inactive students experienced a higher level of procrastination [25]. Comparing to general procrastination, although AP focuses more on students’ procrastination behaviors on academic tasks, it was reasonable to propose the following hypothesis:

**Hypothesis** **1** **(H1).** *PA has a significant and negative impact on AP in college students.*

### 1.2. PA and Self-Efficacy

The positive effects of PA on self-efficacy have been widely recognized. Self-efficacy refers to beliefs in one’s capabilities to organize and execute the courses of action required to produce given attainments [26,27]. Research has found that PA was positively related to self-efficacy in adolescents and older adults [28,29,30]. The positive relationship between PA and self-efficacy was also found in college students, and the higher PA intensity, the higher level of self-efficacy [31]. Additionally, different formats of PA such as aerobic exercise, resistance training, or mind-body technique (e.g., Yoga, Tai Chi) could also enhance self-efficacy [32,33]. Therefore, we proposed the following hypothesis:

**Hypothesis** **2** **(H2).** *PA has a significant and positive impact on self-efficacy in college students.*

### 1.3. Self-Efficacy and AP

Self-efficacy determines individuals’ task initiation and persistence [11]. A low level of self-efficacy is related to behavior avoidance, while a high level of self-efficacy can promote behavior initiation and persistence [26]. Perceiving procrastination as a type of behavior avoidance, research has explored the relationship between self-efficacy and AP, and the negative relationship between the two variables was identified [2,34,35,36]. College students with higher levels of self-efficacy were less likely to experience AP [2,34,35,36]. Therefore, based on the existing literature, we proposed the following hypothesis:

**Hypothesis** **3** **(H3).** *Self-efficacy has a significant and negative impact on AP in college students.*

Since we proposed that PA has a negative impact on AP, and the positive relationship between self-efficacy and AP is supported through the literature and our derived logic, we proposed the following hypothesis:

**Hypothesis** **4** **(H4).** *Self-efficacy significantly mediates the relationship between PA and AP in college students.*

Although previous studies have provided evidence on the relationship between PA and general procrastination, limited attention has been given by scholars in examining the relationship between PA and AP among college students. Moreover, current approaches in decreasing AP found in the literature have been categorized into three focuses, including therapeutic treatment, therapeutic prevention, and instructor/teacher intervention [8]. However, the literature did not emphasize the role of PA in decreasing AP in college students. It is necessary to explore more about the relationship between PA and AP given the numerous physical and mental benefits of PA and its effective role in dealing with procrastination. Furthermore, the lack of a holistic discussion on the relationship between the three variables could limit the theoretical depth of the conclusion in terms of the role of PA in AP, and the specific role of self-efficacy between PA and AP of college students is unknown.

Therefore, this study aimed to further explore the relationship between PA and AP in college students, and clarify the internal relationship between PA, self-efficacy, and AP. Specifically, two questions were answered: (1) What is the impact of PA on AP in college students? (2) Does self-efficacy mediate the relationship between PA and AP? The results of this study would expand the understanding of the importance of PA in decreasing AP so that administrators, policy makers, and practitioners can promote PA in higher education and increase academic success of college students.

## 2. Materials and Methods

### 2.1. Recruitment and Participants

A quantitative survey research method was used in this study. By applying a cross-sectional design, a convenience sampling strategy was employed to recruit participants. Full-time college students enrolled in physical education courses (e.g., table tennis, badminton, aerobics) were selected. A total of 687 undergraduate students (51% males, 49% females) aged 17–23 years (*M* = 19.59, *SD* = 0.89) from a teacher education university in the southeast part of China participated in the study. The sample involved 17% freshmen, 70% sophomores, and 13% juniors. Seniors did not participate in the study as they were not enrolled in any of those physical education courses.

### 2.2. Instruments

In this study, a structured questionnaire was organized to collect college students’ PA, self-efficacy, and AP data. The questionnaire consisted of the following three scales.

#### 2.2.1. Procrastination Assessment Scale-Students (PASS)

The PASS was used to assess student AP levels [37]. The scale includes six domains (academic tasks) (i.e., writing a term paper, studying for an exam, keeping up with weekly reading assignments, performing administrative tasks, reading books borrowed from others or library, and performing academic tasks in general) with a total of 18 items (three items for each domain). For each academic task, subjects indicate on a 5-point Likert scale about the extent to which they procrastinate on the task (1 = never; 5 = always), the extent to which procrastination on the task is a problem for them (1 = never; 5 = always), and the extent they want to decrease their procrastination on the task (1 = never; 5 = always) (Table A1). Since procrastination emphasizes both behavioral delay and psychological distress, scores of the extent to which an individual procrastinates on the task and the extent to which it presents a problem are summed for each academic task (score ranging from 2 to 10) as well as across the six academic tasks (total score ranging from 12 to 60) in order to calculate the AP levels [37]. A higher total score indicates a higher level of AP. 

#### 2.2.2. Physical Activity Rating Scale-3 (PARS-3)

The PARS-3 revised by Liang (1994) was adopted to assess student PA level [38]. The scale included three items focus on PA intensity (what is the intensity of PA that you usually participate in?), PA duration (how long do you spend in each PA session?), and PA frequency (how often do you do PA every month/week?) (Table A2). For each item, five choices were listed with points from 1 to 5 for quantification. For example, the choices for the PA frequency question were less than 1 time/month (1 point), 2 to 3 times/month (2 points), 1 to 2 times/week (3 points), 3 to 5 times/week (4 points), and every day (5 points). The calculation of PA score is based on the following equation: PA score = PA intensity score × (PA duration score − 1) × PA frequency score. The PA score interval ranged from 0 to 100 points. According to the PA score, the PA level was then divided into low, moderate, and high categories: low ≤ 19 points, moderate = 20 to 42 points, and high ≥ 43 points.

#### 2.2.3. Generalized Self-Efficacy Scale (GSES)

The GSES was used to assess student self-efficacy [39]. The scale includes 10 items (e.g., When I am confronted with a problem, I usually find several solutions) that measure individuals’ general beliefs in their competence in effectively dealing with difficult situations (Table A3). A 4-point Likert scale ranging from 1 (strongly disagree) to 4 (strongly agree) was used in the scale. The calculation of self-efficacy score is by summing up all the scores of the 10 items. A high total score would indicate a high level of self-efficacy.

Scales originally written in English (e.g., PASS, GSES) were first translated into Chinese by a bilingual U.S. professor. Then the translated Chinese scales were re-translated into English by another bilingual professor in order to maintain the equivalence and accuracy. The discrepancies were found to be minor. Demographic information including gender, year in university, and major was also included in the questionnaire.

### 2.3. Data Collection

Paper-and-pencil self-administered questionnaires were distributed to students before the start of selected physical education classes. To ensure the quality of responses, the research assistants read the instructions and explained the purposes and requirements of the questionnaire at the beginning. The voluntariness of participation and the confidentiality of responses were also emphasized. Completion of the survey took an average of eight minutes. Students then returned the completed questionnaire at the end of the class.

### 2.4. Data Analysis

SPSS 25.0 software (IBM, Armonk, NY, USA) was used for data analysis. Before analyzing the data, we first calculated the common method variance of the data since the data were obtained by self-reports. Specifically, results from Harman’s one-factor test [40] did not preclude the possibility of common method variance. Specifically, the measurement items of all variables were analyzed by using an unrotated factor analysis. The results indicated that there were six factors that had eigenvalues greater than 1 and the variation explained by the first factors was 28.05%, which was less than the cut-off value of 40%. Therefore, the common method variance is not of great concern and thus was unlikely to confound the interpretations of results in the study.

To ensure that the questionnaire had acceptable reliability and validity for the sample used in this study, confirmatory factor analysis (CFA) was conducted by using Amos 25.0 to examine the construct validity, convergent validity, and discriminant validity of the instrument. Given that PA is an observed variable, the PARS-3 was excluded in CFA, and thus, only PASS and GSES were tested. For construct validity, statistical indices including minimum discrepancy per degree of freedom (CMIN/DF), root mean square error of approximation (RMSEA), goodness-of-fit index (GFI), normed fit index (NFI), and comparative fit index (CFI) were evaluated to examine the model fit of the questionnaire. Indications of a good model fit include a value of 0.08 or less for RMSEA, and a value of 0.95 or more for GFI, NFI, and CFI [41]. In terms of convergent validity, composite reliability (CR) was calculated for every construct, and the cut-off value of CR is 0.6 [42]. Additionally, average variance extracted (AVE) scores were calculated, and AVE scores should be larger than 0.50 in order to have an acceptable convergent validity [43]. However, even if AVE is less than 0.5, but if CR is higher than 0.6, the construct still has an adequate convergent validity [44]. For discriminant validity, $\sqrt{\mathrm{AVE}}$ was calculated. When $\sqrt{\mathrm{AVE}}$ for each of the constructs are higher than the correlations between constructs, it means that the instrument has an acceptable discriminant validity [44]. Additionally, Cronbach’s alpha was calculated by using SPSS 25.0 to assess the reliability of the questionnaire. Acceptable reliability should have an alpha value of 0.70 or above [41].

Descriptive characteristics of college students’ PA, self-efficacy, and AP levels were examined using means, standard deviations (SD), and percentages. To analyze the correlations of the variables, Pearson correlation analysis was first used to test the correlation among college students’ PA level, self-efficacy, and AP, respectively. In addition, linear regression analysis was applied to further examine the relationship among PA level, self-efficacy, and AP. Furthermore, The SPSS macro PROCESS model 4 with 95% confidence interval based on 5000 bootstrap samples [45] was used to examine the indirect effect of PA on AP through self-efficacy. Specifically, the mediation analysis was conducted with PA as the independent variable, AP as the dependent variable, and self-efficacy as the mediation variable. The significance level of all indicators was set as alpha = 0.05.

## 3. Results

### 3.1. Validity and Reliability of the Instrument 

The PASS used in the study had an acceptable reliability with a Cronbach’s α of 0.91. The scale had good model fit for the data, CMIN/DF = 3.18, *p* < 0.001, RMSEA = 0.06, GFI = 0.96, NFI = 0.96, CFI = 0.97. Moreover, the AVE values for each construct were above the 0.5 cut-off value (Table 1), and the $\sqrt{\mathrm{AVE}}$ values for each construct were larger than the correlation between the factors (Table 2), indicating acceptable convergent validity and discriminant validity of the scale.

The GSES used in the study had a Cronbach’s α of 0.85, indicting an acceptable reliability. Additionally, the scale achieved good model fit for the data, CMIN/DF = 2.98, *p* < 0.001, RMSEA = 0.05, GFI = 0.98, NFI = 0.99, CFI = 0.97. The factor loading of GSES ranged from 0.44–0.71. Although the AVE value for the construct was 0.36, it had a CR of 0.85, which was above the cut-off value of 0.6. Therefore, GSES still had an adequate convergent validity.

### 3.2. Correlations of PA, Self-Efficacy, and AP

When interpreting the results, it is necessary to note that we only measured a general self-efficacy level in college students, and not focused on their self-efficacy toward specific competencies or abilities. The average overall PA score was 28.66 (*SD* = 22.81), indicating that college students in the study participated in a moderate level of PA. In terms of self-efficacy, they had an average total score of 24.16 (*SD* = 4.70), which states that they tended to believe their capability in dealing with different situations, although the level was not high. The average score of AP (*M* = 33.51, *SD* = 8.23) presents that college students tended to have some procrastination behaviors in academic tasks (Table 3). The results in Table 3 also present that PA had a significant positive correlation with self-efficacy in college students (*r* = 0.228, *p* < 0.01). In addition, PA was negatively correlated to AP and the relationship was significant (*r* = −0.287, *p* < 0.01). Likewise, significantly negative correlation was also found between self-efficacy and AP (*r* = −0.304, *p* < 0.01).

### 3.3. Mediating Effect of Self-efficacy in PA and AP

**Figure 1:**
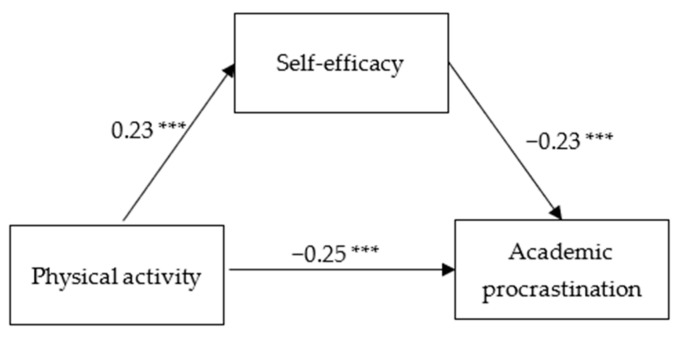
The mediating effect of PA on AP through self-efficacy. Note: *** *p* < 0.001.

The mediation analysis results in Table 4 present that PA significantly predicts AP, and the prediction remains significant even when self-efficacy was entered. In addition, both the direct effect of PA on AP and the mediating effect of self-efficacy had bootstrap confidence intervals (95%) with no zero between their lower and upper limits (Table 5). This finding may suggest that PA not only can directly predict AP, but may also predict AP indirectly through self-efficacy (Figure 1). The direct effect (−0.091) and the mediating effect (−0.019) respectively accounted for 82.70% and 17.30% of the total effect.

## 4. Discussion

The present study further investigated the relationships among PA, self-efficacy, and AP in college students. A simple mediation model was tested to examine the mediating role of self-efficacy in the relationship between PA and AP. All four proposed hypotheses (H1, H2, H3, H4) were accepted. Specifically, PA has a significant and negative impact on AP; PA has a significant and positive impact on self-efficacy; self-efficacy has a significant and negative impact on AP; and self-efficacy significantly mediates the relationship between PA and AP. To the best of our knowledge, this study was the first attempt to examine the relationship between PA and AP with the mediating effect of self-efficacy. The findings of the study provide deeper theoretical insights into the role of PA in dealing with AP in college students.

PA has a significant and negative impact on AP, this finding is consistent with the previous studies on this topic [7,24,25]. In other words, the greater (more time & intensity) PA, the less AP in college students [7,25]. In this study, college students participated in a moderate level of PA in general, and it could significantly decrease student AP behaviors. As suggested by Burka and Lenora (2008), “if you move your body and expand your brain, you can get going on other things you’ve been putting off” [46] (p. 227). Therefore, it is highly recommended that college students participate in a regular PA with moderate-intensity or higher so that the negative impact of PA on AP can be secured. Overall, PA plays a direct and indirect role in decreasing AP in college students. 

The positive impact of PA on self-efficacy in the study is not surprising, and the finding is in line with the previous studies [29,30,31]. Also being consistent with the existing literature, self-efficacy has a negative impact on AP [11,34,35,36]. This study also confirmed the mediating role of self-efficacy in the relationship between PA and AP. In other words, individuals who actively participate in PA are more likely to have a higher level of self-efficacy, which in turn leads to a lower level of AP. Self-efficacy is a strong predictor of performance in academic settings as it is the belief in one’s capabilities to succeed in certain tasks [36]. Students with higher levels of self-efficacy tend to engage more readily in academic tasks [34]. Since participating in regular PA needs individuals’ time management skill [24], the more regular PA they commit to, the more self-confidence they have in managing the time appropriately. Therefore, we believe that the increasing level of self-efficacy in time management as a result of participating in regular PA can potentially decrease the AP behaviors in college students. The rationale was that fewer time management skills results in AP [47]. Besides the mediating role of self-efficacy in the relationship between PA and AP, more mediating or moderating variables can be explored in the future.

There are theoretical and practical implications of the study. This study enriches the inadequate literature on the relationship between PA and AP. The study also highlights the important role of self-efficacy in the relationship between PA and AP, which deepens our understanding of the impact of PA on AP behaviors in college students. Furthermore, the relationships among the three variables presented in this study might help administrators, policy makes, and practitioners understand more about the importance of PA in dealing with college students’ AP behaviors. This can allow actions to be taken to promote regular PA in the higher education setting, and ultimately, to help college students achieve academic success during their years in college.

Although the present study advances our understanding of the relationships among PA, self-efficacy, and AP, some potential limitations should be acknowledged when interpreting the results. First, the findings were generated based on a sample of Chinese college students, so caution needs to be taken when generalizing the results to other populations. Future studies could utilize a random sampling strategy and collect data from different culture backgrounds. Second, due to the cross-sectional design, no causal inference was made. Future studies can use longitudinal designs or experiments to confirm this causality and understand how stable the correlations of the three variables may be throughout time.

Third, this study only measured college students’ general self-efficacy level instead of their self-efficacy toward specific tasks or abilities (e.g., PA or exercise self-efficacy). Future studies could continue to explore the relationship among specific self-efficacy, PA, and AP. Last, it is worth noting that not all empirical studies found a negative relationship between self-efficacy and AP. For example, Žirin (2011) reported no relationship between self-efficacy and AP [6]. Our study did not further explore the reasons for the inconsistent findings. Future research can explore more on the potential moderators that affect the relationship between self-efficacy and AP. For instance, whether the effect is due to cultural differences in the countries where the samples were taken.

## 5. Conclusions

The current study incorporated a psychological mechanism (i.e., self-efficacy) into the relationship between PA and AP in college students. PA had a direct significant and negative impact on AP. Additionally, PA had an indirect significant and negative impact on AP through the mediating role of self-efficacy. Specifically, college students with higher levels of PA reported greater self-efficacy, which in turn contributes to less AP. Thus, PA played a direct and indirect positive role in decreasing AP behaviors in college students. Promoting regular PA is important in higher education to reduce college students’ AP behaviors.

## Figures and Tables

**Table 1:** Convergent validity of PASS.

Constructs	Items	Factor Loading Ranges	CR	AVE
F1	3	0.56–0.88	0.75	0.51
F2	3	0.63–0.90	0.79	0.57
F3	3	0.64–0.88	0.80	0.58
F4	3	0.59–0.92	0.82	0.61
F5	3	0.71–0.86	0.85	0.65
F6	3	0.73–0.87	0.84	0.65

Note: Writing a term paper (F1); studying for an exam (F2); keeping up with weekly reading assignments (F3); performing administrative tasks (F4); reading books borrowed from others or library (F5); performing academic tasks in general (F5); composite reliability (CR); average variance extracted (AVE).

**Table 2:** Discriminant validity of PASS.

	F1	F2	F3	F4	F5	F6
F1	0.72					
F2	0.67	0.75				
F3	0.65	0.67	0.76			
F4	0.59	0.55	0.72	0.78		
F5	0.30	0.23	0.34	0.38	0.81	
F6	0.53	0.54	0.56	0.46	0.43	0.80

Note: Discriminant validity for constructs were the bold numbers ($\sqrt{\mathrm{AVE}})$.

**Table 3:** Correlations of PA, self-efficacy, and AP.

	M	SD	PA	Self-Efficacy	AP
PA	28.66	22.81	-		
Self-efficacy	24.16	4.70	0.228 **	-	
AP	33.51	8.23	−0.287 **	−0.304 **	-

Note: Physical activity (PA); academic procrastination (AP); mean (M); standard deviation (SD); ** *p* < 0.01.

**Table 4:** Mediation analysis results for the three variables.

Regression Equation		Fitting Indices		Regression Coefficient	
Outcome Variables	Predictor Variables	R2	F	β	t
AP		0.09	69.51 ***		
	PA			−0.30	−8.34 ***
Self-efficacy		0.05	37.73 ***		
	PA			0.23	6.14 ***
AP		0.14	56.60 ***		
	PA			−0.25	−6.90 ***
	Self-efficacy			−0.23	−6.31 ***

Note: Physical activity (PA); academic procrastination (AP); *** *p* < 0.001.

**Table 5:** Total effect, direct effect, and indirect effect among the variables.

	Effect Size	Boot SE	Bootstrap 95% CI		Relative Effect Size
			**Lower Limit**	**Upper Limit**	
Total Effect	−0.11	0.01	−0.14	−0.08	100%
Direct Effect	−0.09	0.01	−0.12	−0.06	82.70%
Indirect Effect	−0.02	0.01	−0.03	−0.01	17.30%

Note: Boot Standard Error (Boot SE); Bootstrap 95% Confidence Interval (Bootstrap 95% CI); Relative Effect Size = Direct effect size or indirect effect size divided by total effect size.

## Data Availability

The data in the study are not publicly available in order to protect privacy of the participants.

## Appendix A

**Table A1:** PASS domains and items.

Items	Never	Almost Never	Some-times	Nearly Always	Always
1. To what degree do you procrastinate on writing a term paper?	1	2	3	4	5
2. To what degree is procrastination on writing a term paper a problem for you?	1	2	3	4	5
3. To what extent do you want do decrease your tendency to procrastinate on writing a term paper?	1	2	3	4	5
4. To what degree do you procrastinate on studying for an exam?	1	2	3	4	5
5. To what degree is procrastination on studying for an exam a problem for you?	1	2	3	4	5
6. To what extent do you want do decrease your tendency to procrastinate on studying for an exam?	1	2	3	4	5
7. To what degree do you procrastinate on keeping up with weekly reading assignments?	1	2	3	4	5
8. To what degree is procrastination on keeping up with weekly reading assignments a problem for you?	1	2	3	4	5
9. To what extent do you want do decrease your tendency to procrastinate on keeping up with weekly reading assignments?	1	2	3	4	5
10. To what degree do you procrastinate on performing administrative tasks?	1	2	3	4	5
11. To what degree is procrastination on performing administrative tasks a problem for you?	1	2	3	4	5
12. To what extent do you want do decrease your tendency to procrastinate on performing administrative tasks?	1	2	3	4	5
13. To what degree do you procrastinate on reading books borrowed from others or library?	1	2	3	4	5
14. To what degree is procrastination on reading books borrowed from others or library a problem for you?	1	2	3	4	5
15. To what extent do you want do decrease your tendency to procrastinate on reading books borrowed from others or library?	1	2	3	4	5
16. To what degree do you procrastinate on performing academic tasks in general?	1	2	3	4	5
17. To what degree is procrastination on performing academic tasks in general a problem for you?	1	2	3	4	5
18. To what extent do you want do decrease your tendency to procrastinate on performing academic tasks in general?	1	2	3	4	5

**Table A2:** PARS-3 questions and answers.

Answers	Question 1	Question 2	Question 3
	What is the intensity of PA that you usually participate in during the past month?	How long do you spend in each PA session?	How often do you do PA every month/week?
1	Light-intensity physical activity (go for a walk and radio broadcast exercises, etc.)	Less than 10 min	Less than 1 time/month
2	Light- to moderate-intensity physical activities (recreational volleyball, table tennis, jogging and Tai Chi, etc.)	11–20 min	2–3 times/month
3	Moderate- to vigorous-intensity physical activities (cycling, running, and table tennis, etc.)	21–30 min	1–2 times/week
4	Vigorous but not lasting physical activities with sweating a lot (badminton, volleyball, basketball, tennis, and football, etc.)	31–59 min	3–5 times/week
5	Vigorous and lasting physical activities with sweating a lot (racing, aerobics practicing, and swimming, etc.)	More than 60 min	Every day

**Table A3:** GSES items.

Items	Strongly Disagree	Disagree	Agree	Strongly Agree
1. I can always manage to solve difficult problems if I try hard enough.	1	2	3	4
2. If someone opposes me, I can find means and ways to get what I want.	1	2	3	4
3. It is easy for me to stick to my aims and accomplish my goals.	1	2	3	4
4. I am confident that I could deal efficiently with unexpected events.	1	2	3	4
5. Thanks to my resourcefulness, I know how to handle unforeseen situations.	1	2	3	4
6. I can solve most problems if I invest the necessary effort.	1	2	3	4
7. I can remain calm when facing difficulties because I can rely on my coping abilities.	1	2	3	4
8. When I am confronted with a problem, I can usually find several solutions.	1	2	3	4
9. If I am in a bind, I can usually think of something to do.	1	2	3	4
10. No matter what comes my way, I am usually able to handle it.	1	2	3	4
